# Characterization of the alternative splicing landscape in lung adenocarcinoma reveals novel prognosis signature associated with B cells

**DOI:** 10.1371/journal.pone.0279018

**Published:** 2023-07-11

**Authors:** Ming-Ming Shao, Kan Zhai, Zhong-Yin Huang, Feng-Shuang Yi, Sheng-Cai Zheng, Ya-Lan Liu, Xin Qiao, Qing-Yu Chen, Zhen Wang, Huan-Zhong Shi

**Affiliations:** Department of Respiratory and Critical Care Medicine, Beijing Institute of Respiratory Medicine and Beijing Chao-Yang Hospital, Capital Medical University, Beijing, China; University of Crete & IMBB-FORTH, GREECE

## Abstract

**Background:**

Lung cancer is the second most commonly diagnosed cancer and the leading cause of cancer-related death. Malignant pleural effusion (MPE) is a special microenvironment for lung cancer metastasis. Alternative splicing, which is regulated by splicing factors, affects the expression of most genes and influences carcinogenesis and metastasis.

**Methods:**

mRNA-seq data and alternative splicing events in lung adenocarcinoma (LUAD) were obtained from The Cancer Genome Atlas (TCGA). A risk model was generated by Cox regression analyses and LASSO regression. Cell isolation and flow cytometry were used to identify B cells.

**Results:**

We systematically analyzed the splicing factors, alternative splicing events, clinical characteristics, and immunologic features of LUAD in the TCGA cohort. A risk signature based on 23 alternative splicing events was established and identified as an independent prognosis factor in LUAD. Among all patients, the risk signature showed a better prognostic value in metastatic patients. By single-sample gene set enrichment analysis, we found that among tumor-infiltrating lymphocytes, B cells were most significantly correlated to the risk score. Furthermore, we investigated the classification and function of B cells in MPE, a metastatic microenvironment of LUAD, and found that regulatory B cells might participate in the regulation of the immune microenvironment of MPE through antigen presentation and promotion of regulatory T cell differentiation.

**Conclusions:**

We evaluated the prognostic value of alternative splicing events in LUAD and metastatic LUAD. We found that regulatory B cells had the function of antigen presentation, inhibited naïve T cells from differentiating into Th1 cells, and promoted Treg differentiation in LUAD patients with MPE.

## Introduction

Lung cancer is the second most commonly diagnosed cancer and the leading cause of cancer-related death, with an estimated 2.21 million new cases and 1.80 million lung cancer-related deaths worldwide in 2020 [1]. Lung cancer is a highly aggressive form of cancer and has a 5-year survival rate of less than 20% [2]. Lung adenocarcinoma (LUAD) is the most common form of lung cancer, accounting for approximately 50%–60% of all lung cancer cases.

Malignant pleural effusion (MPE) is commonly encountered among LUAD patients and is a common cause of morbidity worldwide [3,4]. The appearance of MPE means that the primary tumor has spread systematically, resulting in a decrease in the life expectancy and quality of life of patients [5–7]. Previously, tumor-induced lymphatic obstruction was thought to lead to MPE formation; however, in recent years, several studies have demonstrated that interactions between tumor cells and the host immune system and vasculature result in increased net fluid production due to enhanced plasma extravasation into the pleural space [8,9]. The importance of T cells in tumor immunity has been extensively studied and is well established. Our previous studies have demonstrated that several subsets of CD4^+^ T cells, including regulatory T cells (Tregs), Th17, Th22, and Th9 cells, play important immune regulatory roles in the pathogenesis of MPE [10–15].

Compared to T cells, the potential contributions of B cells to tumor immunity are less well investigated. Apart from the production of antibodies (Abs), B cells can shape the functions of other immune cells by secreting cytokines, presenting antigens, and providing co-stimulation in tumor immunity [16,17]. A subset of IL-10 competent regulatory B cells (Bregs) have been functionally identified by their ability to express cytoplasmic IL-10 in mice [18,19] and humans [20,21]. Several lines of evidence have shown that Bregs play an important role in human tumor immunity. Peripheral counts of Bregs cells were found to be significantly higher in patients with lung cancer [22]. However, the specific mechanism remains to be studied.

Aberrant expression of RNA-processing genes (RPGs) is an important hallmark of tumors [23], especially those encoding splicing factors (SFs), which govern the alternative splicing events of genes. Several SFs, including SRSF1, SF3B1, UPF1, and RBM10, have been shown to be involved in regulating the malignant phenotype of lung cancer and are related to prognosis, indicating that alternative splicing events are involved in the carcinogenesis, progression, and metastasis of tumors [24–26].

In the present study, we used data on SFs, alternative splicing events, and the tumor immune microenvironment to conduct a comprehensive analysis of prognostic factors in LUAD, especially in metastatic LUAD patients. We constructed a risk signature based on 23 alternative splicing events and identified it as an independent prognostic indicator. This risk score had better predictive performance in metastatic patients, among all patients. We found that B cells in the infiltrating lymphocytes were most significantly correlated to the risk score and that Bregs might participate in the regulation of the immune microenvironment of MPE through antigen presentation and promotion of Treg differentiation.

## Materials and methods

### mRNA-seq and alternative splicing events in The Cancer Genome Atlas data

The mRNA-seq data of LUAD patients in The Cancer Genome Atlas (TCGA) and corresponding clinical data (filtered for an overall survival time of more than 30 days) were downloaded from UCSC Xena (https://xena.ucsc.edu/). Finally, 504 tumor samples and 60 control samples were selected for subsequent analysis. Alternative splicing data measured by the percent spliced in (PSI) value (the percentage of samples with PSI was more than 75%) were collected from SpliceSeq [27] (http://bioinformatics.mdanderson.org/TCGASpliceSeq).

### Identification of prognosis-related signatures and construction of a risk model

We performed univariate Cox regression analyses to identify survival-related genes or alternative splicing events with *P* < 0.05 in LUAD in the TCGA database. The Least Absolute Shrinkage and Selection Operator (LASSO) model was used to obtain the minimum number of genes to establish the risk model. Next, the selected candidate genes were used to establish a multivariate Cox model. The risk score was calculated as follows: risk score = Ʃ(*b*_*i*_ × *Exp*_*i*_), where *b*_*i*_ represents the weight of the respective signature, and *Exp*_*i*_ represents the expression value.

### Patients and specimens

The study protocol was approved by the Institutional Review Board for human studies of Beijing Chaoyang Hospital, Beijing, China (No. 2017-ke-57); and informed written consent was obtained from each subjects. From April 2019 through March 2021, the patients with undiagnosed pleural effusions were admitted to the department of respiratory and critical care medicine of Beijing Chaoyang Hospital for diagnostic investigation. The patients were included subsequently if the examinations of pleural fluid and/or biopsy specimens established a definite diagnosis of MPE.

The pleural fluid was collected in heparin-treated tubes from each subject, using a standard thoracocentesis technique within 24 h after hospitalization. Mononuclear cells from pleural fluid were isolated by Ficoll-Hypaque gradient centrifugation (MP Biomedicals, Solon, OH) within 1 h, followed by resuspension with phosphate-buffered saline.

### Flow cytometry

CD19^+^ B cells were identified by flow cytometry after surface or intracellular staining with human-specific Abs conjugated with FITC, PE, APC, PE-cy7, PerCP-cy5.5, APC-cy7, BV421, or BV510. These human Abs included anti-CD3, anti-CD8, anti-CD19, anti-CD24, anti-CD25, anti-CD27, anti-CD40, anti-CD80, anti-CD86, anti-TGF-β, anti-TNF-α, anti-CCR3, anti-CXCR3, anti-IL-10, anti-IL-17, anti-IFN-γ, anti-Foxp3, anti-Viability Dye, and anti-IgD, which were purchased from Thermo Fisher (San Jose, CA, USA) or Thermo Fisher (Carlsbad, CA, USA). Appropriate species-matched Abs served as isotype controls. Flow cytometry was performed on a FACS Canto II (BD Biosciences), and data were analyzed using BD FCS Diva Software and FCS Express 5 plus software (De Novo Software, Los Angeles, CA, USA).

### Determination of protein concentration

The concentration of IL-10, TGF beta and TNF alpha protein were tested by enzyme-linked immunosorbent assay (ELISA) kits with reference to the manufacturer’s specifications (Thermo Fisher Scientific, US; Abcam, UK). All samples were assayed in duplicate.

### Cell isolation and culture

Human mononuclear cells were isolated from MPE through density gradient centrifugation. Bregs and naïve B cells were separated from mononuclear cells using FACS Aria II (BD Biosciences). CD4^+^CD25^‒^ naïve T cells were isolated from mononuclear cells using a naïve CD4^+^ T cell isolation kit II (Miltenyi Biotech) and then cultured at 37°C in 5% CO_2_ in 96-well plates (2 × 10^5^ cells per well) in 200 μL of RPMI-1640 medium with 10% FBS. Purified Bregs and naïve B cells from MPE were cultured with naïve CD4^+^ T cells in the ratio 1:1 for 5 days with plate-bound anti-CD3 (5 μg/mL) and anti-CD28 mAb (2 μg/mL).

### Differential expression analysis and enrichment analysis

Filter out genes with Fragments Per Kilobase of exon model per Million mapped fragments value < 0.01 in 80% of the samples. The Limma package (3.48.3) was used to calculate the differential expression genes and alternative splicing events between tumor with normal samples, and the differentially expression genes were characterized as FDR < 0.05 and absolute value of fold change > 2. Gene ontology (GO) and Kyoto Encyclopedia of Genes and Genomes (KEGG) enrichment analysis was performed by clusterProfiler (4.0.5) and org.Hs.eg.db (3.13.0) package with differentially expressed genes.

### Transcriptome analysis

1 × 106 naïve B cells and Bregs were isolated by FACS Aria II from MPE. A total amount of 3 μg RNA per sample was purified from total RNA using poly-T oligo-attached magnetic beads. Then PCR was performed with Phusion High-Fidelity DNA polymerase, Universal PCR primers, and Index (X) Primer. The clustering of the index-coded samples was performed on a cBot Cluster Generation System using TruSeq PE Cluster Kit v3-cBot-HS (Illumia) according to the manufacturer’s instructions. The raw sequence data reported in this paper are available at the GSA for Human of the Beijing Institute of Genomics, Chinese Academy of Sciences (GSA for Human: HRA001450).

### Statistical analysis

Data are expressed as mean ± SD. Parametric tests were used since the data of CD19^+^ B cells and their subsets were normally distributed, as determined by a normality test. Comparisons of data between different groups were performed using Student’s *t*-test. Comparisons of B cell subsets in pleural fluid and in the corresponding blood were made using a paired *t*-test. Analysis was conducted using SPSS version 23.0 Statistical Software (Chicago, IL, USA) and R (4.1.0). A *P*-value of <0.05 was considered as statistically significant.

## Results

### Expression of RPGs and their biological function in LUAD

**Fig 1:**
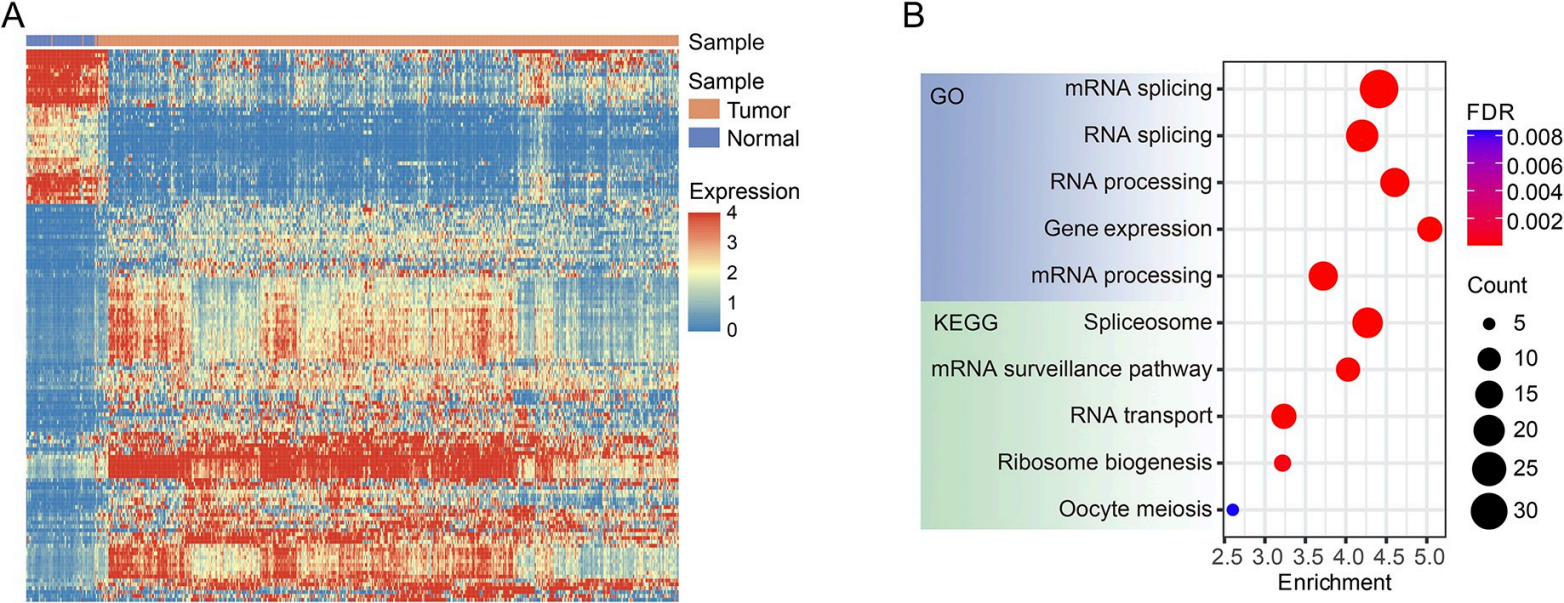
Expression of RNA-processing genes in LUAD (lung adenocarcinoma) in The Cancer Genome Atlas (TCGA) database. (A) Heatmap showing the expression profile of 134 RNA-processing genes in LUAD and control tissues. All these 134 genes were associated with patient survival. (B) Gene ontology and Kyoto Encyclopedia of Genes and Genomes enrichment analyses of 134 survival-related RNA-processing genes in LUAD patients.

Altered expression of RPGs, which participate in and regulate mRNA and protein expression, results in altered activities of cancer-related genes. We evaluated the prognostic effects of 907 RPGs collected from the Molecular Signatures Database (S1 Table) and identified 134 genes associated with LUAD patient survival in the TCGA cohort, including 93 genes with a hazard ratio (HR) of more than 1 and 41 genes with an HR of less than 1. Among these 134 genes, 95 genes were abnormally expressed in tumor tissues, of which 69 genes were significantly upregulated and 26 genes were significantly downregulated in tumor tissues (Fig 1A). Of the highly expressed genes, 84% (58/69) were associated with poor patient prognosis, while this proportion was only 38% among the lowly expressed genes. Since these RPGs participate in multiple processes, from transcription to mRNA maturation, we performed enrichment analysis to identify the most important biological processes in which these survival-related RPGs were enriched. Both GO and KEGG analyses indicated that these genes were mainly involved in RNA splicing pathways (Fig 1B).

### Prognostic signature building of RNA SFs

**Fig 2:**
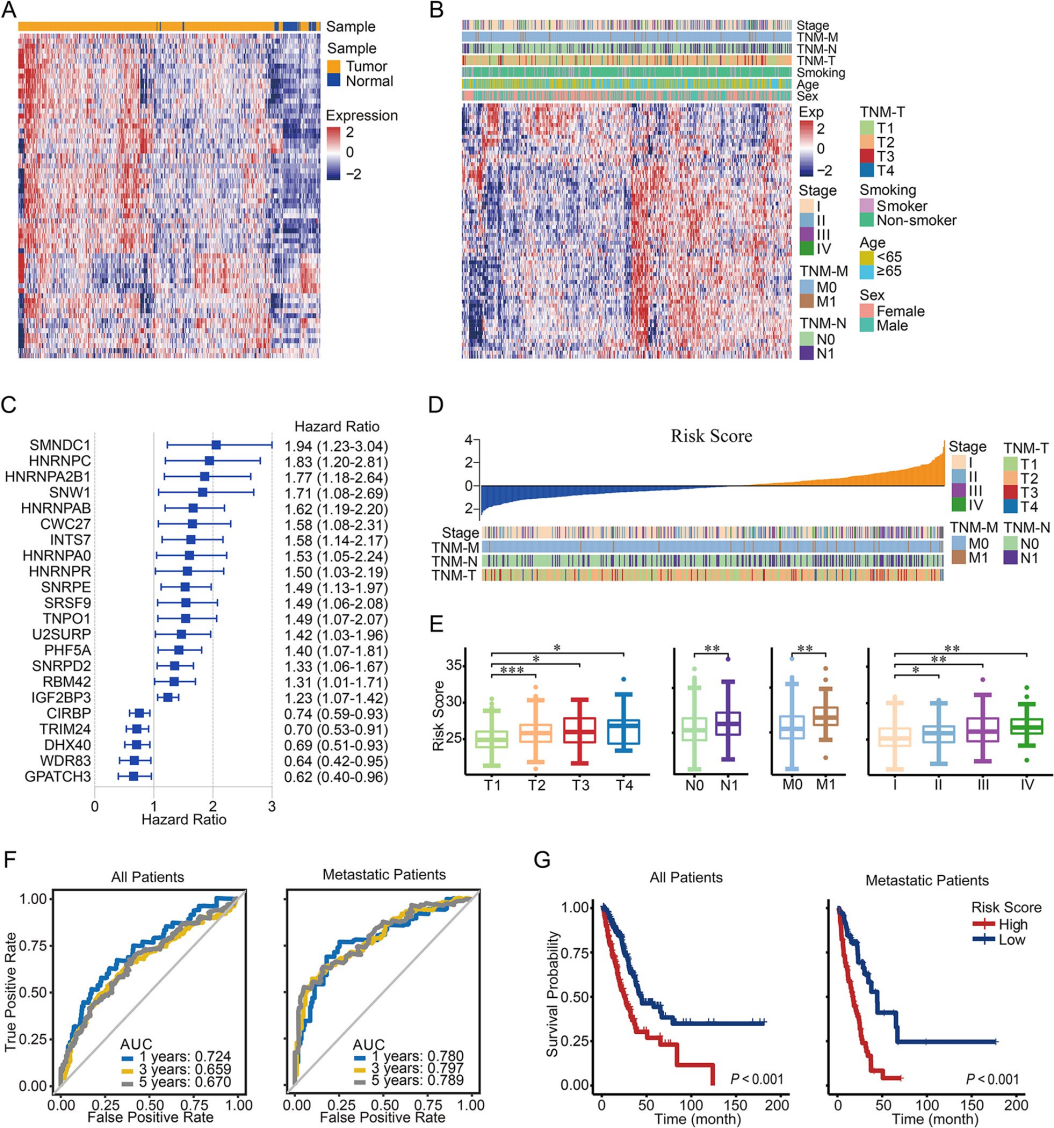
Expression profile of survival-related splicing factors in LUAD in the TCGA database. (A) Heatmap showing the expression profile of 65 survival-related splicing factors in LUAD and control tissues. (B) Heatmap showing the expression profile of 65 survival-related splicing factors in LUAD tissues and clinical characteristics. (C) Forest plot of hazard ratios for 22 survival-related splicing factors included in the SF-based risk score. For all multivariate Cox regression analyses, *P* < 0.05 was considered to indicate significance. (D) Tumor stages and TNM classifications sorted based on the risk score in ascending order. (E) Risk score sorted based on TNM classification and tumor stage. (F) ROC curves of risk scores for 1-, 3-, and 5-year survival in LUAD patients in the TCGA database. Left panel, all patients. Right panel, metastatic patients. (G) Kaplan–Meier overall survival curves of all patients (left panel) and metastatic patients (right panel) grouped by risk score.

Given that alternative splicing is an important process, in order to categorize clinical outcomes of LUAD patients, we collected 382 SFs from SpliceAid2 and analyzed their expression and their correlation with patient survival in the TCGA cohort. Univariate Cox hazard analysis identified 65 genes associated with patient survival, of which 49 genes were significantly aberrantly expressed in tumor tissues, especially in advanced tumor tissues (Fig 2A and 2B and S2 Table). To efficiently stratify the clinical outcomes of LUAD patients with SFs, we applied the LASSO Cox regression algorithm to the 65 genes in the TCGA dataset. A total of 22 genes with non‐zero coefficients were selected to build the risk score (Fig 2C). Patients with advanced tumor stage (stage II–IV or T2–4) and metastatic tumors (N1 and M1) had higher risk scores than others (Fig 2D and 2E). Receiver operating characteristic (ROC) curve analysis showed that the signature risk score had a good predictive value for the 1-year, 3-year, and 5-year survival of LUAD patients, especially in patients with tumor metastasis. The area under the ROC curve (AUC) values of the risk score for 1-, 3-, and 5-year survival were 72.4%, 65.9%, and 67.0%, respectively, in all patients and were 78.0%, 79.7%, and 78.9%, respectively, in metastatic patients (Fig 2F). Patients with low risk scores had significantly longer overall survival than patients with high risk scores, among both metastatic patients and all patients (Fig 2G).

### Building a prognostic signature of alternative splicing events

**Fig 3:**
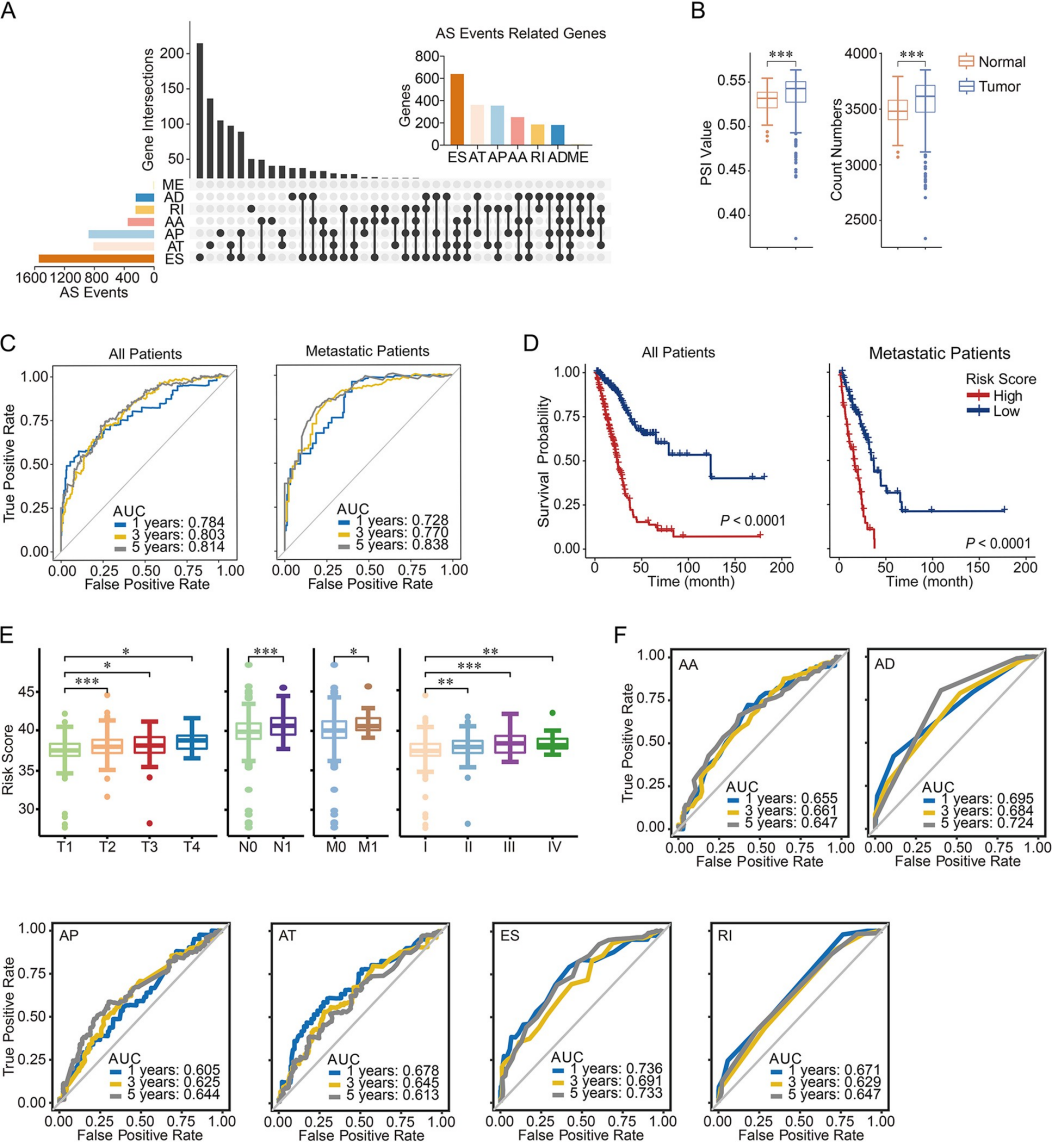
Survival-related alternative splicing events in LUAD patients in the TCGA database. (A) UpSet plot of alternative splicing events and the corresponding genes. (B) PSI values (left panel) and counts (right panel) of tumor and control tissues. (C) ROC curves of risk scores for 1-, 3-, and 5-year survival in LUAD patients in the TCGA database. Left panel, all patients. Right panel, metastatic patients. (D) Kaplan–Meier overall survival curves of all patients (left panel) and metastatic patients (right panel) grouped by risk score. (E) Risk score of each TNM classification and tumor stage. (F) ROC curves of risk scores for 1-, 3-, and 5-year survival in LUAD patients in the TCGA database for each alternative splicing event type. AA, alternative acceptor site; AD, alternative donor site; AP, alternative promoter; AT, alternative terminator; ES, exon skipping; RI, retained intron; ME, mutually exclusive exon.

The SF-based model, with AUC values of 0.670 in all patients and 0.789 in metastatic patients for 5-year survival were not satisfactory. Therefore, we next examined whether alternative splicing events could be used to build a more effective risk model. To this end, 43,949 alternative splicing events associated with 10,367 genes were found in TCGA LUAD patients (Fig 3A). Exon skipping was the most common alternative splicing event, accounting for more than one-third of the total events, and mutually exclusive exons were the least common alternative splicing event. PSI values and numbers of alternative splicing events were both higher in tumor tissues than in control tissues (Fig 3B). Similarly, we established a risk score model through LASSO regression and multivariate Cox analysis. We were surprised to find that the predictive value for the survival of LUAD patients and metastatic patients was higher than that of the SF-based model, especially for long-term survival (Fig 3C and S3 Table). In total, 43 alternative splicing events passed our Lasso regression and multivariate survival analysis, including 18 exon skipping, 10 alternative promoters, 5 alternative donor site, 4 alternative acceptor sites, 3 alternative terminators, and 3 retained intron events. The AUC values of the risk score were 78.4%, 80.3%, and 81.4% in all patients and 72.8%, 77.0%, and 83.8% in metastatic patients for 1-, 3-, and 5-year survival, respectively. Similar to the SF-based risk score, patients with low risk scores had significantly longer overall survival than patients with high risk scores in both metastatic patients and all patients (Fig 3D). Patients with advanced tumor stage (stage II–IV or T2–4) and metastatic tumors (N1 and M1) had higher risk scores than others (Fig 3E). We also calculated the AUC values for all alternative splicing event types separately and found that their predictive values for survival were not as high as that of the model based on all events (Fig 3F). Due to the small number of mutually exclusive exon alternative splicing events, no survival analysis was performed for this type. The univariate and multivariate Cox analyses revealed that the alternative splicing risk score was an independent prognostic factor in LUAD patients (Table 1).

**Table 1:** The univariate and multivariate Cox analysis of risk score and clinical features.

Clinical feature	Univariate analysis			Multivariate analysis		
	HR	95% CI	P value	HR	95% CI	P value
Risk score	6.85	(3.17–8.95)	<0.001	2.93	(2.01–4.28)	<0.001
Sex	1.10	(0.81–1.51)	0.575			
Age						
< 65y	1 (reference)			1 (reference)		
≥ 65y	1.01	(1.00–1.02)	0.030	1.02	(1.01–1.04)	<0.001
Smoking						
Non-smoker	1 (reference)					
Smoker	1.08	(0.68–1.71)	0.751			
Stage						
I	1 (reference)			1 (reference)		
II	2.58	(1.75–3.79)	<0.001	2.13	(1.11–4.10)	0.024
III	3.18	(2.09–4.84)	<0.001	1.96	(0.89–4.33)	0.094
IV	3.16	(1.66–6.03)	<0.001	2.26	(1.04–4.90)	0.040
T						
T1	1 (reference)			1 (reference)		
T2	1.60	(1.09–2.34)	0.016	1.40	(0.62–1.38)	0.691
T3	2.54	(1.40–4.60)	0.002	0.92	(0.48–2.01)	0.959
T4	3.02	(1.27–7.18)	0.012	0.98	(0.46–3.10)	0.722
N						
N0	1 (reference)			1 (reference)		
N1	2.60	(1.89–3.59)	<0.001	1.40	(0.80–2.40)	0.250
M						
M0	1 (reference)			1 (reference)		
M1	1.95	(1.05–3.60)	0.034	1.50	(0.81–3.16)	0.202

### Correlation of the alternative splicing-related risk score with B cell infiltration in LUAD

**Fig 4:**
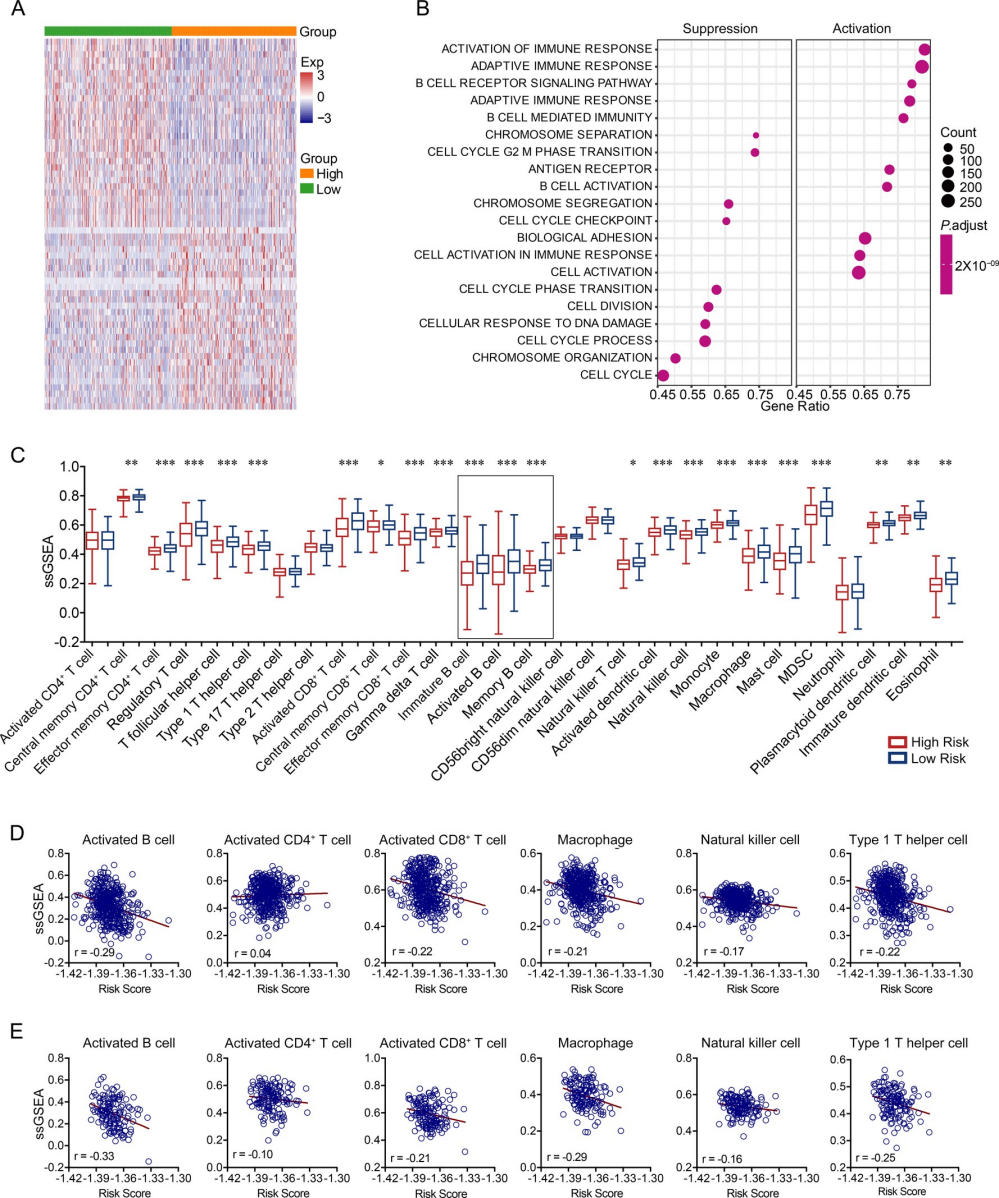
Tumor-infiltrating lymphocytes in LUAD patients with low or high risk scores. (A) Heatmap of differentially expressed genes between the high and low risk score groups. (B) GO enrichment analyses of differentially expressed genes between the high and low risk score groups. (C) Single-sample GSEA of tumor-infiltrating immune cells. (D) Correlation of risk score and single-sample GSEA of six types of tumor-infiltrating immune cells in all patients. (E) Correlation of risk score and single-sample GSEA of six types of tumor-infiltrating immune cells in metastatic patients.

We divided patients into high and low risk score groups according to the median value of the alternative splicing-based risk score. We characterized the RNA expression profiles in both groups. In addition to malignancy-related biological processes such as the cell cycle, cell division, and DNA stability, the expression of genes related to activation of the immune response, especially the adaptive immune response and B cell activation, was significantly different between the two groups (Fig 4A and 4B). Due to the infiltration of large numbers of immune cells in the tumor microenvironment, to explore whether there were differences in immune status between the two groups, we performed single sample gene set enrichment analysis (GSEA) to analyze the proportion of infiltrating immune cells in LUAD [28]. The results showed that the immune infiltration ratio of the high risk score group was lower than that of the low risk group; immature B cells, activated B cells, and memory B cells were the top three cell types with the largest fold change between the two groups (Fig 4C). Next, we used Spearman correlation to analyze the correlation between the risk score and infiltrating immune cells. Activated B cells had the highest correlation coefficient among the immune cells; activated B cell counts were higher in metastatic patients (Fig 4D and 4E). All these results indicated that the function and proportion of tumor-infiltrating B cells were correlated with the alternative splicing risk score, especially in metastatic patients. Based on this, we wanted to investigate whether multivariate analysis combining risk score and B-cell infiltration fraction would enhance the predictive power of patient survival. We first divided patients into high and low groups according to the median activated B-cell infiltration fraction. Among all patients, patients with high activated B-cell infiltration fraction had significantly longer overall survival than patients in low group. However, activated B-cell infiltration fraction was not associated with patient survival in metastatic patients (S1A Fig). Next, we combined risk score and activated B-cell infiltration score to analyze the impact on patient survival. Possibly due to the significant correlation between these two, the effect on patient survival was not significantly improved compared with the use of risk scores alone (S1B Fig).

### Distribution and characteristics of Bregs and naïve B cells in MPE

**Fig 5:**
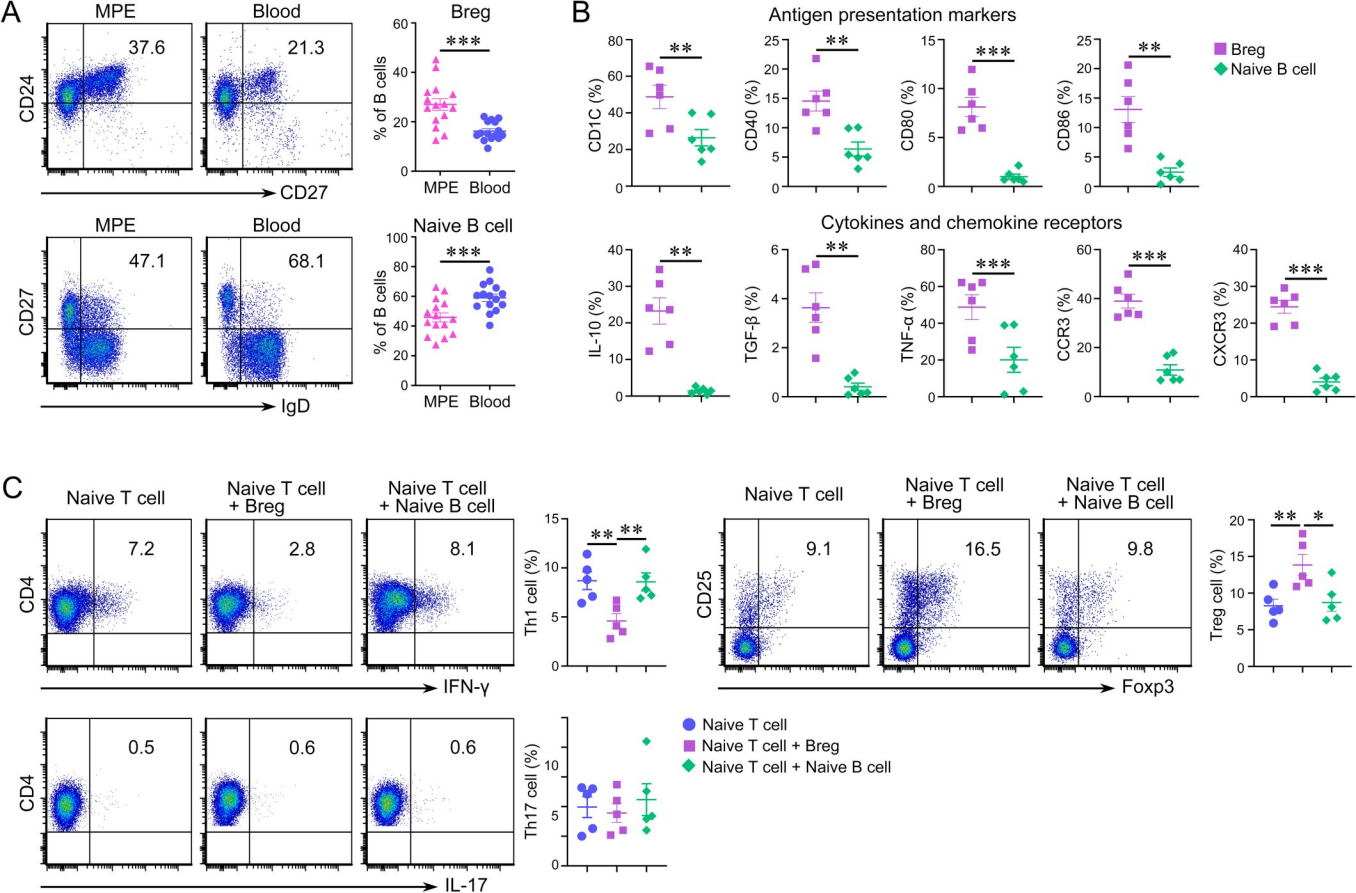
Distribution and characteristics of regulatory B cells (Bregs) and naïve B cells in malignant pleural effusion (MPE). Expression of CD19, CD24, CD27, and IgD was detected using flow cytometry. Bregs were characterized as CD19^+^CD24^hi^CD27^+^, and naïve B cells were defined as CD19^+^CD27^‒^IgD^+^ cells. (A) Representative flow cytometry dot plot and percentages of Bregs and naïve B cells in MPE and blood (***P < 0.001; paired Student’s *t*-test). (B) Characteristics of Bregs and naïve B cells in MPE, as determined by flow cytometry (**P < 0.01, ***P < 0.001, paired Student’s *t*-test). (C) Bregs inhibited Th1 cell differentiation but promoted Treg differentiation in vitro and had no effect on Th17 cells (**P < 0.01; one-way ANOVA).

Next, in order to identify the role of B cells in the microenvironment of metastatic tumors, we examined the proportions and functions of B cell subtypes in LUAD-related MPE, an important type of lung cancer metastasis. We observed an increase of CD19^+^CD24^hi^CD27^+^ Bregs in MPE compared with that in blood, while the CD19^+^CD27^‒^IgD^+^ naïve B cells showed a significant decrease compared with that in blood (Fig 5A). By comparing the characteristics of Bregs and naïve B cells in MPE by flow cytometry and ELISA, we found that the expression levels of antigen presentation markers (CD1C, CD40, CD80, and CD86) and cytokine and chemokine receptors (IL-10, TGF-β, TNF-α, CCR3, and CXCR3) were all significantly upregulated in Bregs (Figs 5B and S2). Simultaneously, Bregs from MPE inhibited Th1 cell differentiation but promoted Treg differentiation and had no effect on Th17 cells in vitro (Fig 5C). These results indicated that Bregs might play an important regulatory role in the tumor microenvironment of MPE.

### Differential expression profile of Bregs and naïve B cells

**Fig 6:**
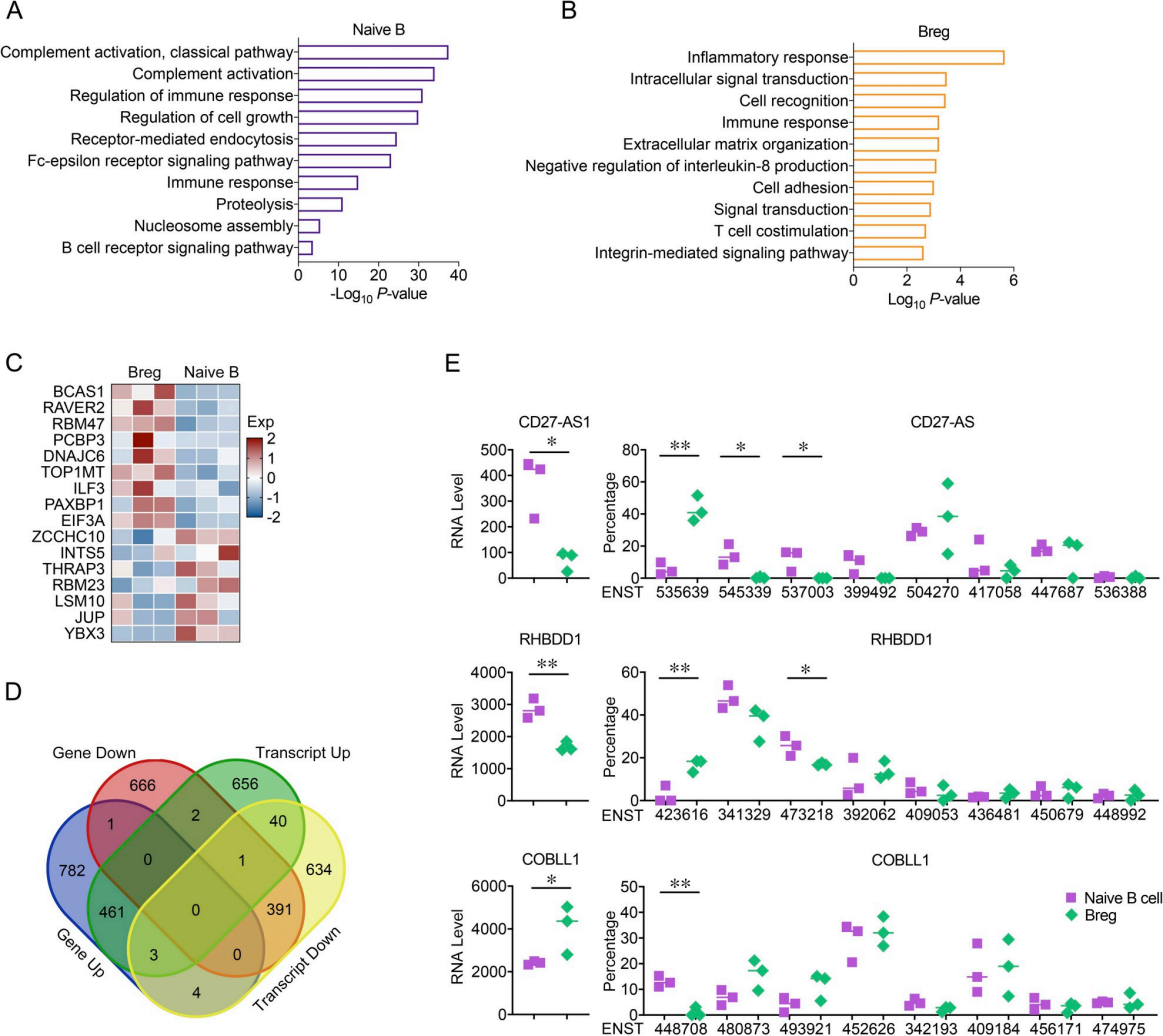
Differentially expressed genes and transcripts in Bregs and naïve B cells. (A) GO enrichment analyses of differentially expressed genes that were upregulated in naïve B cells. (B) Gene Ontology enrichment analyses of differentially expressed genes that were upregulated in Bregs. (C) Heatmap of SFs in Bregs and naïve B cells. (D) Venn diagram showing the numbers of differentially expressed genes and differentially expressed transcripts between Bregs and naïve B cells. (E) RNA expression levels (left panel) and transcript expression levels (right panel) of CD27-AS1, RHBDD1, and COBLL1 in Bregs and naïve B cells.

To gain further insight into the function of Bregs, we performed RNA sequencing analysis in naïve B cells and Bregs isolated from the MPE to investigate the differential expression gene pattern. GO enrichment analysis revealed that the top enriched pathways in Bregs were the inflammatory response and the immune response (Fig 6A and 6B). Moreover, the SFs BCAS1, RAVER2, RBM47, PCBP3, DNAJC6, TOP1MT, ILF3, PAXBP1, and EIF3A were also upregulated in Bregs (Fig 6C). The RNA sequencing results showed 1251 upregulated genes, 1061 downregulated genes, 1163 upregulated transcripts, and 1037 downregulated transcripts in Bregs (Fig 6D). Different transcripts of the same gene might be expressed separately, and sometimes, the gene expression level alone cannot reflect the difference in the transcriptome between two cell types. For example, CD27-AS1 had the highest proportion of the transcript ENST00000504270 in naïve B cells, while the highest proportion in Bregs was ENST00000535639. For the *RHBDD1* gene, the transcript ENST00000341329 was expressed in more than 50% of naïve cells, while it was expressed in less than 40% of Bregs. ENST00000423616 was expressed in approximately 20% of Bregs, while it was almost not expressed in naïve B cells. Similarly, the ENST000000448708 transcript of the *COBLL* gene was expressed in more than 10% of naïve B cells, but not in Bregs (Fig 6E). We additionally selected genes with differences in alternative splicing events between Bregs and naïve B cells. Through enrichment analysis, we found that these genes were mainly involved in signaling pathways such as regulation of T cell differentiation and positive regulation of interleukin-4/5/13 production. This further illustrated the significant immune function differences between Bregs and naïve B cells (S3 Fig).

## Discussion

We described the expression patterns and correlation of SFs, alternative splicing events, clinical characteristics, and immunologic features in LUAD using TCGA data. We identified the SFs and alternative splicing events significantly associated with LUAD patient survival and built a risk score based on alternative splicing events, which had a higher predictive value than the SF-based model. This risk score predicted the prognosis of metastatic LUAD patients more effectively than that of all LUAD patients. We further analyzed tumor-infiltrating lymphocytes in the low and high risk score groups and identified B cells as the tumor-infiltrating lymphocytes with the highest correlation with risk score, especially in metastatic patients. Moreover, we systematically analyzed Bregs, a B cell subset that was enriched in MPE (the typical lung cancer metastasis microenvironment), and identified important alternative splicing events among them.

B cells play an important role in immune regulation during the development of MPE. Our previous research revealed that in an MPE mouse model, B cell knockout promoted MPE formation by inhibiting CD4^+^ cell proliferation. Moreover, it was found that naïve B cells accounted for the highest proportion of B cells in MPE, and functionally activated naïve B cells could regulate the Th1/Th17 balance [29]. However, the specific function of Bregs, another subgroup of B cells, in MPE has not been evaluated. In the present study, we found that the proportion of CD19^+^CD24^hi^CD27^+^ Bregs in MPE was significantly higher than that in blood, while the proportion of naïve B cells was higher in peripheral blood. Therefore, we hypothesized that Bregs might play a different role than naïve B cells in MPE formation. CD1C molecules are expressed on antigen-presenting cells and provide T lymphocytes with certain foreign lipids that affect both innate and adaptive immune responses [30]. CD40 binds to CD40L on T cells and plays an important role in T cell activation and differentiation. Previous studies have shown that CD80 and CD86 synergistically constrain the Th1 response with IL-10 in human IL-10^+^ Bregs [31,32]. The upregulation of CD1C, CD40, CD80, and CD86 on Bregs shown in our study indicated that Bregs might exhibit a strong ability of antigen presentation in the MPE immune microenvironment in vivo due to tumor antigen stimulation.

Chemokines and chemokine receptors are involved in multiple immunological processes. Bregs express IL-10, TGF-β, and TNF-α in most inflammatory and tumor microenvironments [33–36]. Our findings suggested that the expression of IL-10, TGF-β, TNF-α, CXCR3, and CCR3 was significantly higher in Bregs in the MPE microenvironment than in naïve B cells. These results showed that Bregs might be affected by chemotaxis from the peripheral circulation to the pleural cavity and might participate in the regulation of MPE development by secreting cytokines, including IL-10, TGF-β, and TNF-α.

Our previous studies revealed that Th cells and Tregs were recruited into the MPE microenvironment [37,38]. Th1 and Th17 cells could regulate each other in a mouse model of MPE. Th1 cells inhibit Th17 differentiation and promote MPE formation by secreting IFN-γ, while Th17 cells inhibit Th1 differentiation and MPE formation and improve mouse survival by producing IL-17. This study showed that Bregs in MPE inhibited Th1 cell differentiation but promoted Treg differentiation and had no effect on Th17 cells in vitro. Taken together, these findings suggested that Bregs may regulate the tumor immune microenvironment by inhibiting naïve T cells from differentiating into Th1 cells and promoting Treg differentiation.

Alternative splicing events of individual genes can regulate the biological functions of cells [39,40]. Antisense RNA could form a duplex structure with sense mRNA and modify mRNA stability and translation efficiency [41]. Evidence demonstrated that antisense RNA plays a crucial role in both tumor progression and the immune response [42–45]. Most Bregs are characterized by high expression of CD27 [20,21]. It is reasonable to speculate that CD27-AS1 has an important function in regulating the expression of CD27. Among the CD27-AS1 transcripts, ENST00000535639, which is an alternative splicing form produced by intron retention, accounts for about 40% of the total transcripts in Breg, while there was almost no such transcript in naïve B cells. We speculate that this full-length transcript of ENGT00000535639 might have a weaker effect on the degradation of CD27 mRNA, thereby further reducing the level of functional CD27-AS1 in Bregs and increasing the expression of CD27.

Researchers have constructed LUAD prognostic models using immune scores, metabolism-associated genes, or ferroptosis-related genes and have achieved favorable predictive efficacy [46–48]. In our study, we found that the alternative splicing-based model had a better prognostic value in patients with metastatic tumors than in all patients.

There are some limitations in our study. First, our alternative splicing-based prognostic risk score requires validation in an independent external cohort. Second, we found that Bregs isolated from MPE can inhibit Th1 cell differentiation and promote Treg differentiation; future studies are required to elucidate the effects of Bregs on other cells like CD8^+^ T cells or macrophages in MPE.

In summary, we evaluated the prognostic value of alternative splicing events in LUAD and metastatic LUAD. We revealed that Bregs had the function of antigen presentation, inhibited naïve T cells from differentiating into Th1 cells, and promoted Treg differentiation in LUAD patients with MPE. Alternative splicing of CD27-AS1 in Bregs and naïve B cells might explain the high expression of CD27 in Bregs. Although the depth of our study is limited, we provide a new perspective on alternative splicing events in Bregs that extends the field of LUAD and MPE research.

## Supporting information

S1 Fig Survival analysis with the fraction of active B cell infiltration in LUAD. (A) Kaplan–Meier overall survival curves of all patients (left panel) and metastatic patients (right panel) grouped by activated B cell infiltration. (B) The Kaplan–Meier overall survival curves of TCGA LUAD patients grouped by the gene signature of risk score and activated B cell infiltration fraction. The high and low groups are divided by the median value of the mean expression of risk score or activated B cell infiltration fraction. The significant was calculated using the two-sided log-rank test.
(PDF)

S2 Fig Expression of IL-10, TGF-β and TNF-α was detected using enzyme linked immunosorbent assay. Bregs were characterized as CD19^+^CD24^hi^CD27^+^, and naïve B cells were defined as CD19^+^CD27^‒^IgD^+^ cells. **P < 0.01, ***P < 0.001, paired Student’s *t*-test.
(PDF)

S3 Fig Gene ontology enrichment analyses of genes with differences in alternative splicing events between Bregs and naïve B cells. (PDF)

S1 Table RNA processing gene. (XLSX)

S2 Table Univariate Cox regression analyses of RNA splicing factors. (XLSX)

S3 Table Hazard ratios and multivariate Cox P value for 23 survival-related alternative splicing events involved in risk score. (XLSX)

S1 File Extended materials and methods section. (DOCX)
