# Supplementary figures and images for: Characterization of the alternative splicing landscape in lung adenocarcinoma reveals novel prognosis signature associated with B cells
Source: PLoS One. 2023 Jul 11;18(7):e0279018. doi: 10.1371/journal.pone.0279018 (PMC10335703; doi:10.1371/journal.pone.0279018)

**A**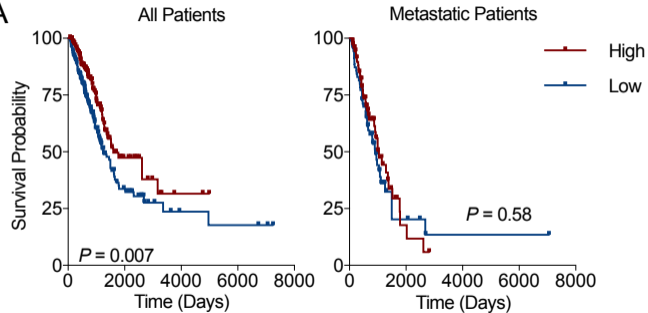**B**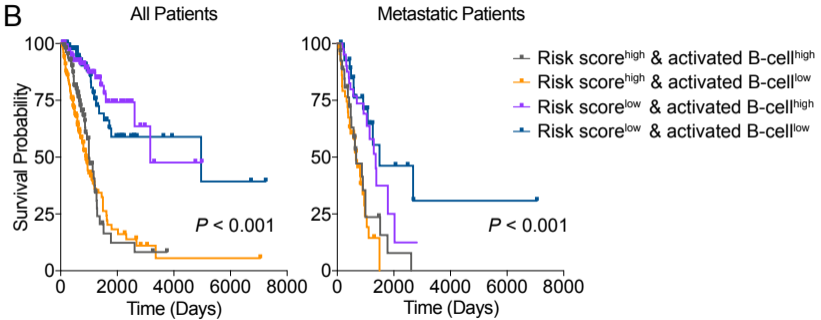

Supplement: S1 Fig — (A) Kaplan–Meier overall survival curves of all patients (left panel) and metastatic patients (right panel) grouped by activated B cell infiltration. (B) The Kaplan–Meier overall survival curves of TCGA LUAD patients grouped by the gene signature of risk score and activated B cell infiltration fraction. The high and low groups are divided by the median value of the mean expression of risk score or activated B cell infiltration fraction. The significant was calculated using the two-sided log-rank test. (PDF) [file pone.0279018.s001.pdf]

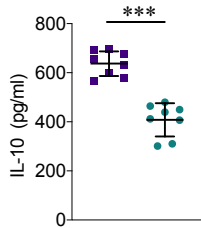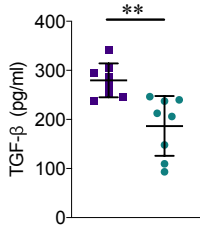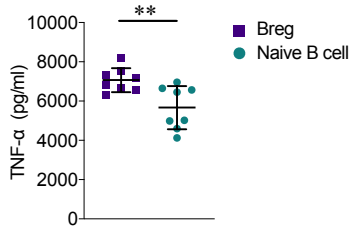

Supplement: S2 Fig — Bregs were characterized as CD19+CD24hiCD27+, and naïve B cells were defined as CD19+CD27‒IgD+ cells. **P < 0.01, ***P < 0.001, paired Student’s t-test. (PDF) [file pone.0279018.s002.pdf]

## Go Enrichment Analysis

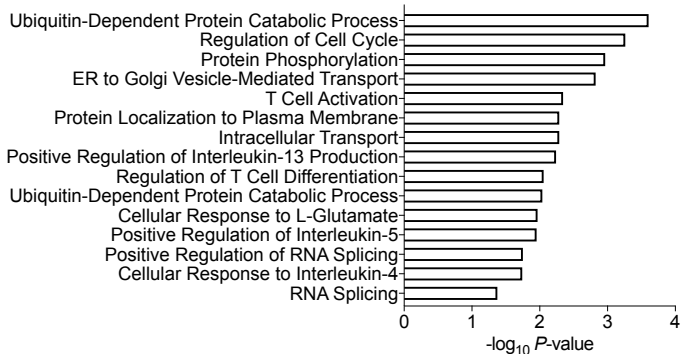

Supplement: S3 Fig — (PDF) [file pone.0279018.s003.pdf]
