# Supplementary material for: Characterization of the alternative splicing landscape in lung adenocarcinoma reveals novel prognosis signature associated with B cells
Source: PLoS One. 2023 Jul 11;18(7):e0279018. doi: 10.1371/journal.pone.0279018 (PMC10335703; doi:10.1371/journal.pone.0279018)
Supplement: S1 File — (DOCX) [file pone.0279018.s007.docx]

**Materials and methods**

**Flow cytometry Antibodies**

APC Anti-Human CD3 Thermo Fisher 17-0038-42

FITC, Anti-Human CD8 Thermo Fisher 11-0088-42

PercP-cy5.5 Anti-Human CD19 BD Biosciences 561295

PE-cy7 Anti-Human CD24 BD Biosciences 561646

PE-cy7 Anti-Human CD25 BD Biosciences 557741

APC Anti-Human CD27 BD Biosciences 558664

BV421 Anti-Human CD40 BD Biosciences 563396

BV421 Anti-Human CD80 BD Biosciences 564160

PE Anti-Human CD86 BD Biosciences 560957

BV421 Anti-Human TGF-β BD Biosciences 562962

APC Anti-Human TNF-α Thermo Fisher 48-7349-42

PE Anti-Human CCR3 BD Biosciences 558165

PE Anti-Human CXCR3 BD Biosciences 557185

PE Anti-Human IL-10 Thermo Fisher 12-7108-82

PercP-cy5.5 Anti-Human IL-17 Thermo Fisher 45-7179-42

PE-cy7 Anti-Human INF-γ Thermo Fisher 25-7319-82

PE Anti-Human FOXP3 Thermo Fisher 12-4777-42

eFluor 450 Fixable Viability Dye 65-0863-18

eFluor 780 Fixable Viability Dye 65-0865-14

FITC Anti-Human IgD BD Biosciences 555778

ELISA Kit

Human IL-10 ELISA Kit Abcam ab185986

Human TNF-alpha ELISA Kit Abcam ab285312

TGF beta-1 Human/Mouse Uncoated ELISA Kit Invitrogen 88-8350-88
